# The Non-Protein Amino Acid BMAA Is Misincorporated into Human Proteins in Place of l-Serine Causing Protein Misfolding and Aggregation

**DOI:** 10.1371/journal.pone.0075376

**Published:** 2013-09-25

**Authors:** Rachael Anne Dunlop, Paul Alan Cox, Sandra Anne Banack, Kenneth John Rodgers

**Affiliations:** 1 Cell Biology Group, School of Medical and Molecular Biosciences, University of Technology Sydney, Ultimo, New South Wales, Australia; 2 Institute for Ethnomedicine, Jackson Hole, Wyoming, United States of America; Macquarie University, Australia

## Abstract

Mechanisms of protein misfolding are of increasing interest in the aetiology of neurodegenerative diseases characterized by protein aggregation and tangles including Amyotrophic Lateral Sclerosis (ALS), Alzheimer’s disease (AD), Parkinson’s disease (PD), Lewy Body Dementia (LBD), and Progressive Supranuclear Palsy (PSP). Some forms of neurodegenerative illness are associated with mutations in genes which control assembly of disease related proteins. For example, the mouse sticky mutation *sti*, which results in undetected mischarging of tRNA^Ala^ with serine resulting in the substitution of serine for alanine in proteins causes cerebellar Purkinje cell loss and ataxia in laboratory animals. Replacement of serine 422 with glutamic acid in tau increases the propensity of tau aggregation associated with neurodegeneration. However, the possibility that environmental factors can trigger abnormal folding in proteins remains relatively unexplored. We here report that a non-protein amino acid, β-N-methylamino-L-alanine (BMAA), can be misincorporated in place of l-serine into human proteins. We also report that this misincorporation can be inhibited by l-serine. Misincorporation of BMAA into human neuroproteins may shed light on putative associations between human exposure to BMAA produced by cyanobacteria and an increased incidence of ALS.

## Introduction

Protein translation is a highly efficient and accurate process for assembling the 20 standard amino acids into proteins. Error rates in translation are relatively rare (1 in 10^3^ to 10^4^) and rely on the ability of the system to discriminate between the 20 protein (or canonical) amino acids [1]. However such rare errors which result in the mischarging of tRNA by the wrong amino acid can result in misfolded or truncated proteins, and subsequent cell damage, such as in the sti (sticky) mutation in mice where serine is substituted for alanine, resulting in neurodegeneration [2]. A mutation causing glutamic acid to be substituted for serine at position 422 in tau increases the likelihood of tau aggregation associated with neurodegeneration [3].

During protein translation the genetic code is interpreted from information contained in the nucleic acid sequence in messenger RNA (mRNA) into the primary amino acid sequence of a polypeptide chain. Fidelity of protein synthesis, at the translational level, relies on the specificity of protein amino acid and cognate tRNA recognition by tRNA synthetases. In certain cases when two protein amino acids have a similar structure, a proofreading step checks the structural fidelity of an amino acid to the catalytic site of the tRNA synthetase and the bond is hydrolyzed if the wrong amino acid is attached [1]. Hundreds of non-protein amino acids exist; many occur naturally in plants, some are produced *in vivo* from amino acid oxidation, and others are synthetically produced [3]–[8]. Non-protein amino acids that are close structural analogues of any of the 20 protein amino acids can bind to an amino acid-tRNA synthetase and become mistakenly peptide bonded into a polypeptide chain [8]. In order to be incorporated into proteins, non-protein amino acids have to be present in the cell at sufficient concentrations to successfully compete with the protein amino acid. Examples of non-protein amino acids that are misincorporated into proteins include canavanine and 3,4 dihydroxyphenylalanine (l-dopa) [6], [8]–[10].

β-N-methylamino-l-alanine (BMAA) has been found to be associated with proteins in cyanobacteria and other organisms [11]–[13]. The possibility that BMAA is misincorporated into proteins was bolstered by a study in which autoradiographic analysis was performed on mice after a single injection of ^3^H-BMAA producing “a distribution pattern similar to that of a protein forming amino acid” [14]. ^3^H-BMAA uptake was demonstrated in tissues with high levels of protein synthesis, and radioactivity was maintained in the tissue after acid extraction [14]. These data are consistent with incorporation of BMAA by protein synthesis.

## Materials and Methods

MRC-5 cells, a human lung fibroblast cell line, were from American Tissue and Cell Culture (ATCC, Virginia, USA). SH-SY5Y cells, a human neuroblastoma cell line, were from the European Collection of Cell Cultures (ECACC, Public Health England, UK). Human umbilical vein endothelial cells (HUVECs) were obtained from umbilical cords as described previously [15]. L-BMAA [methyl-3H] (^3^H-BMAA) (80 Ci/mmol, 0.5 m Ci/mL) was obtained from American Radiolabeled Chemicals and L-[4,5-3H]leucine (139 Ci/mmol and 1 mCi/mmol) was purchased from Amersham. Dulbecco’s modification of Eagle’s minimum essential medium (DMEM) and HAMS F12 were from JRH Biosciences, (Lenexa, Kansans, USA). BMAA, dithiothreitol (DTT), all amino acids, cycloheximide (CHX), sodium dodecyl sulphate (SDS) and pronase E (protease from *Streptomyces griseus*) were from Sigma Chemical Co. (Sigma-Aldrich, Castle Hill, NSW, Australia). Bicinchoninic acid (BCA) assay protein reagent was from Pierce Biotechnology (Rockford, IL, USA). The BD Pharminigen™ Annexin V-FITC apoptosis detection kit was from BD Biosciences (Sydney, Australia). Water was from a Milli Q 4 stage system (Millipore-Waters, Lane Cove NSW, Australia). Other chemicals and solvents were AR grade.

### Cell Culture

MRC-5 cells, (passage number 14–19), and SH-SY5Y cells, (passage number 30–32), were maintained in DMEM, or DMEM/Hams F12 respectively containing 10 % fetal bovine serum (FBS), 4 mM l-glutamine, 100 U/mL penicillin, and 100 µg/mL streptomycin at 37°C in a humidified atmosphere of 5% CO_2_ and 95% air. HUVECs were harvested enzymatically with type II collagenase (Sigma-Aldrich) under sterile conditions as described by Minter [16] and established as primary cell cultures in M199 (Trace Biosciences, Sydney, Australia) containing 20% FBS, 4 mM L-glutamine, 0.5% endothelial cell growth promoter, 100 U/mL penicillin, and 100 µg/mL streptomycin. All media were prepared with endotoxin-free water (Baxter) and filtered with Zetapore filters (Cuno Life Sciences Division). Cells were seeded at 3×10^5^ cells per well in 6 well plates and allowed to adhere overnight (16 hours) before treatment.

### Studies Examining Incorporation of BMAA into Proteins and Inhibition of Incorporation by Cycloheximide and Amino Acids

MRC-5 cells were incubated with ^3^H-BMAA (31.25 nM) in Hank’s buffered salt solution (HBSS) containing 10% FBS. After 2, 4, and 16 hours, cells were washed three times in phosphate buffered saline (PBS) supplemented with 10% FBS and lysed by freeze thawing three times in Triton X-100 (0.1%). The protein concentration in the lysate was determined using the BCA assay [17], radiolabel in the cell lysate quantified by liquid scintillation counting (LSC) and the amount of radiolabel present in proteins expressed as disintegrations per minute (DPM) per cell protein. Cell proteins were then isolated by trichloroacetic acid (TCA) (10%) precipitation washed three times in TCA (10%), and dissolved in formic acid (neat). To determine if incorporation of radiolabel into proteins was protein synthesis dependent, MRC-5, SH-SY5Y and HUVEC cells were incubated with ^3^H-BMAA (31.25 nM) with or without CHX 2 µg/ml for 16 hours. The amount of radiolabel present in cell proteins in the CHX-treated cultures was expressed relative to that of control cultures, which was set at 100%. Parallel cultures of MRC-5 cells were incubated with ^3^H-leucine (41 nM) with or without CHX 2 µg/ml under identical culture conditions and processed as before.

To examine the ability of individual amino acids to reduce incorporation or radiolabel into proteins, MRC-5 cells were incubated with ^3^H-BMAA (31.25 nM) for 16 hours in the presence of individual amino acids (250 µM) and the radiolabel present in the cell proteins assessed as before. All of the 20 protein amino acids (l-isomers) were examined individually in triplicate cell cultures. To confirm that l-serine had an inhibitory effect on incorporation of radiolabel into proteins, MRC-5 cells were incubated with ^3^H-BMAA (31.25 nM) for 16 hours in the presence of l-serine (0, 50, 100 and 250 µM) and in a separate experiment with 250 µM l-serine and d-serine. Incorporation of radiolabel was determined relative to cells incubated in medium containing no serine. MRC-5 cells were then incubated with ^3^H-BMAA (31.25 nM) for 16 hours in HBSS alone, HBSS containing all 20 protein amino acids (400 µM) or 19 protein amino acids expect l-serine and radiolabel in cell protein assessed as before.

### Recovery of Radiolabel from Cell Proteins Generated by Incubating SH-SY5Y Cells with ^3^H-BMAA

SH-SY5Y cells were incubated with ^3^H-BMAA (31.25 nM) for 24 hours and cell proteins isolated by TCA (10%) precipitation. Proteins were washed three times in TCA (10%), rinsed in ice-cold acetone and re-dissolved in PBS. The amount of radiolabel released from the proteins (i.e. not TCA precipitable) after incubation at 37°C with DTT (1 mM) and SDS (2%) with DTT (DTT/SDS) was determined by LSC. Cell proteins were also incubated with pronase (2 mg/mL) for 48 hours in 100 µM Tris hydrochloric acid (HCl) buffer pH 8 containing 20 mM CaCl_2_ at 37°C or in HCl (12 M) for 12 hours and the release of radiolabel quantified relative to that of the buffer alone (for pronase) or water (for HCl). All protein samples were processed in triplicate.

### Recovery of Incorporated BMAA from Proteins Following Hydrolysis

After incubation with ^3^H-BMAA or non-labeled BMAA, cells were washed three times with PBS, lysed by freeze thawing in Triton X-100 (0.1%) and cell proteins precipitated in TCA (10%). Protein pellets were washed three times with TCA (10%) and hydrolyzed in boiling 6 M HCl at 110°C for 16 hours as previously described [18]. Protein pellets were freeze-dried and reconstituted in 20 mM HCl. Particulates were removed by centrifugation through 0.22 µM membranes. Non-labeled BMAA was detected in total lysate by derivatization with 6-aminoquinolyl-N-hydroxysuccinimidyl carbamate (AQC, Waters Corporation, Australia) and quantified by a validated liquid chromatography tandem mass spectrometry (LC/MS/MS) method (see below). ^3^H-BMAA was quantified as described previously [19].

### Liquid Chromatography Tandem Mass Spectrometry

Hydrolyzed samples as above were resuspended in 20 mM HCl. All samples were appropriately diluted for a balanced reaction before being derivatized with AQC and checked by dilution series and by evaluating double derivatized lysine relative to single derivatized lysine. Samples were analyzed using a triple quadrupole, heated electrospray ionization tandem mass spectrometer (HESI-MS/MS) instrument (Thermo Scientific Finnigan TSQ Quantum UltraAM, San Jose, CA) after separation with an Ultra High Pressure Liquid Chromatography (Waters Acquity-UHPLC) system with a Binary Solvent Manager, Sample Manager and a Waters AccQTag Ultra C18 column (part# 186003837, 2.1×100 mm) at 55°C.

Separation was achieved using gradient elution at 0.65 ml/min in aqueous 0.1% (v/v) formic acid (Eluent A) and 0.1% (v/v) formic acid in acetonitrile (Eluent B): 0.0 min = 99.1% A; 0.5 min = 99.1% A curve 6; 2 min = 95% A curve 6; 3 min = 95% A curve 6; 5.5 min = 90% A curve 8; 6 min = 15% A curve 6; 6.5 min = 15% A curve 6; 6.6 min = 99.1% A curve 6; 8 min = 99.1% A curve 6. Nitrogen gas was supplied to the HESI probe with a nebulizing pressure of 40 psi and a vaporization temperature of 400°C. The tandem mass spectrometer was operated under the following conditions: the capillary temperature was set at 270°C, capillary offset of 35, tube lens offset of 110, auxiliary gas pressure of 35, spray voltage 3500, source collision energy of 0, and multiplier voltage of -1654. A divert valve was used to deflect flow during the beginning and end of the gradient. The second quadrupole was pressurized to 1.0 mTorr with 100% argon. Product-ion analysis of BMAA used *m/z* 459 as the precursor ion for collision-induced dissociation (CID) and thereby all other ions were excluded in the first quadrupole. Further two-step mass filtering was performed during selective reaction monitoring (SRM) of BMAA after CID in the second quadrupole, monitoring the following transitions: *m/z* 459 to 119, collision energy (CE) 21 eV; *m/z* 459 to 171 CE 38 eV; *m/z* 459 to 188 CE 38 eV; *m/z* 459 to 214 CE 35 eV; *m/z* 459 to 258 CE 21 eV; and *m/z* 459 to 289 CE 17 eV. The resultant product ions were detected within the third quadrupole and their relative abundances were quantified. Chromatographic separation with unique retention times, and unique product ions, and ion ratios confirmed the identification of BMAA relative to any possible isomers [2,4-diaminobutryic acid (DAB) and N-(2-aminoethyl)glycine][20].

Detection limits (LOD) and limits of quantification (LOQ) of BMAA on the LC-MS/MS were determined experimentally by injecting a dilution series of authenticated stock solutions at 4 concentrations (0.015, 0.15, 1.5, 15 µg/l). The EPA Method Detection Level (MDL) was used which defines the LOD as the minimum concentration of substance that can be measured and reported with 99% confidence that the analyte concentration is greater than zero. The MDL (48 femtomoles) was calculated using the standard deviation of replicates multiplied by the t statistic with α = 0.01 and n = 1 degrees of freedom. The LOQ (0.48 picomoles) was calculated by multiplying the MDL by 10. For validation purposes, the standard curve for BMAA had the following equation: f(x) = 31887463155463x + 19212, r^2^ = 0.99). The intra-day precisions for the transition *m/z* 459 to 171 (n = 8) were evaluated according to the percent relative standard deviation (RSD) for peak area (mean 3018539, 6.3 RSD) and retention time (mean 4.98 min, 0.21 min RSD). The intra-day single reaction monitoring ratio (SRM) of *m/z* 459 to 289 and *m/z* 459 to119 relative to *m/z* 459 to 171 (n = 9) was 29.5 +/− 2.0 SD and 18.4 +/− 1.2 SD respectively. The inter-day precision for the transition *m/z* 459 to 171 (n = 8) was evaluated for peak area (mean 2484745, 5.0 RSD) and retention time (mean 5.02 min, 0.26 min RSD). The inter-day SRM ratio of *m/z* 459 to *m/z* 289 and *m/z* 459 to *m/z* 119 relative to *m/z* 459 to *m/z* 171 (n = 21) was 30.7 +/− 2.4 SD and 17.2 +/− 1.5 SD respectively. Samples were run blinded to the instrument operator as well as the lab staff.

### Autofluorescent Imaging of Cells

MRC-5 cells were incubated in DMEM supplemented with 300 µM BMAA in the presence or absence of 300 µM l-serine for 96 hours with daily medium changes. Cellular autofluorescence was visualized using an inverted fluorescent microscope (Olympus IX71) as described previously [21].

### Lactate Dehydrogenase (LDH) Assay

LDH release from cells was determined as described previously [22].

### Binding of Annexin V to Phosphatidylserine (PS) Exposed on the Plasma Membrane

Apoptosis or necrosis was measured by simultaneous staining with propidium iodide (PI) and Annexin V using the BD Pharminigen™ Annexin V–FITC apoptosis detection kit and flow cytometry performed as described previously [23].

### Statistical Analysis

Statistical comparisons were made using Student’s unpaired two tailed T-tests in GraphPad Prism, version 4.0c. *P*<0.05 was considered significant.

## Results

### Incorporation of BMAA into Human Proteins was Protein Synthesis Dependent

**Figure 1:**
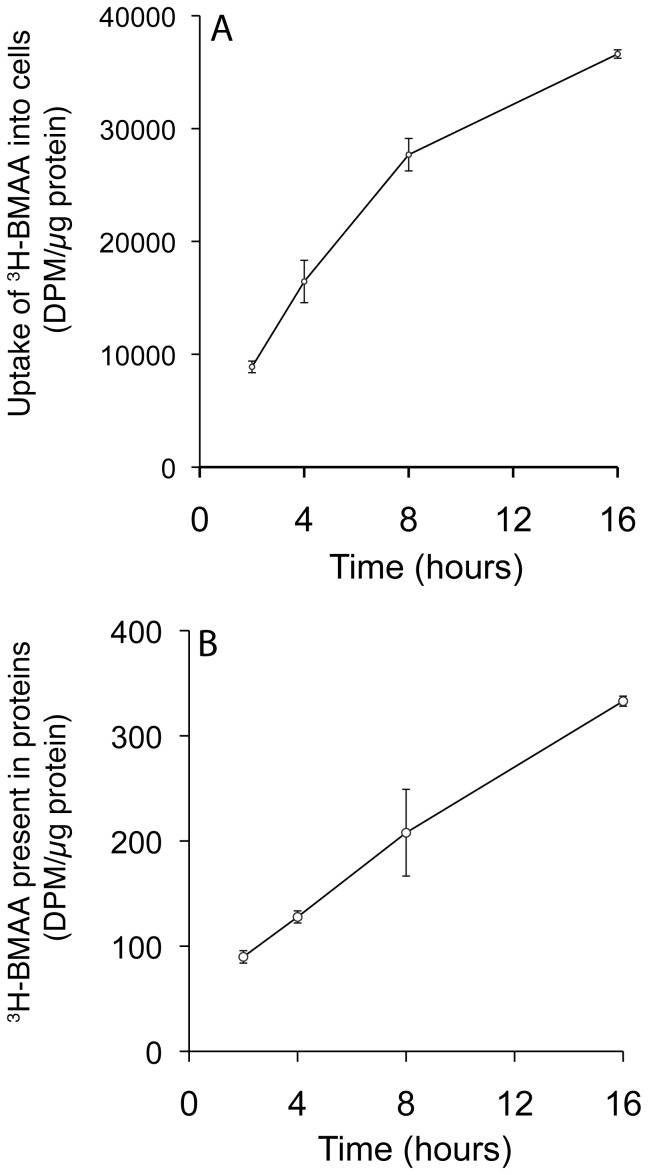
Uptake and incorporation of ^3^H-BMAA into proteins by MRC-5 cells. Panel A, uptake of radiolabel by cells was expressed as disintegrations per minute (DPM) per µg of cell protein. Panel B, radiolabel in the cell protein fraction was expressed as DPM per µg of total cell protein. Values are mean +/− SD for three independent experiments (n = 3).

**Figure 2:**
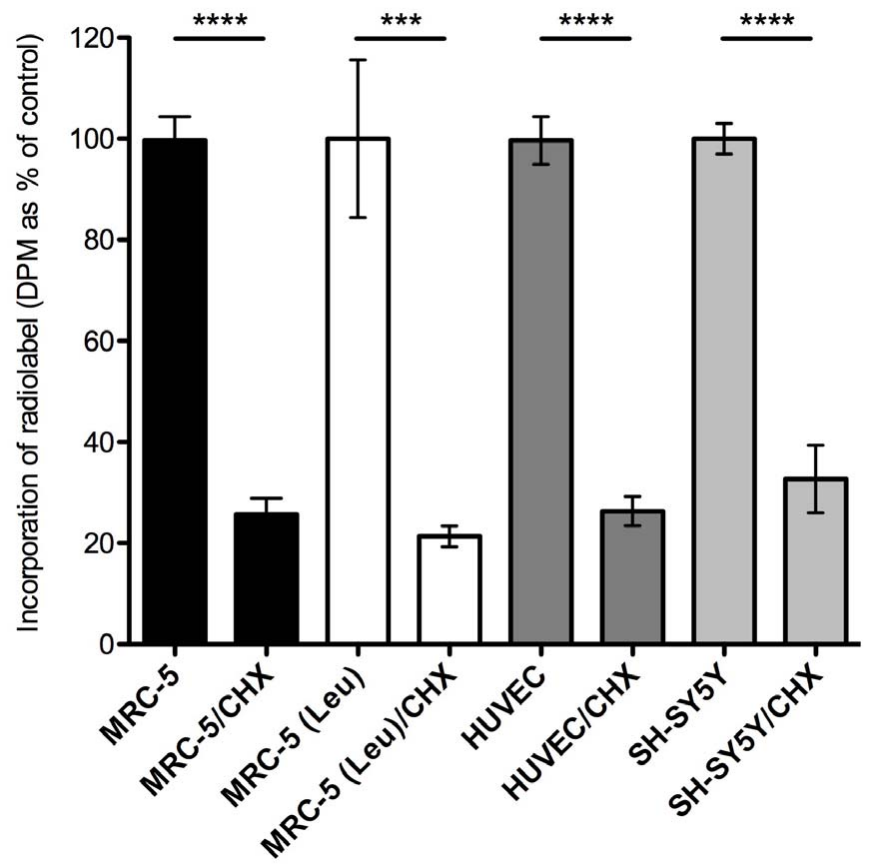
Inhibition of incorporation of radiolabel into cell protein by cycloheximide. MRC-5, HUVEC and SH-SY5Y cells were incubated with ^3^H-BMAA with or without CHX (2 µg/ml). Cells proteins were isolated and the amount of radiolabel present in proteins (DPM per cell protein) determined and expressed as a percentage of control where control treatments were designated 100%. Parallel cultures of MRC-5 cells were incubated with ^3^H-leucine (41 nM) with or without CHX and the effects of CHX similarly determined (open bars). Values are mean +/− SD for three independent experiments (n = 3). Radiolabel incorporation in each cell type was compared with and without CHX using Student’s two-tailed t-test (***P<0.001 ****P<0.0001).

Incubation of human MRC-5 fibroblasts with ^3^H-BMAA in culture medium depleted in amino acids resulted in a time-dependent increase in radiolabel in cell lysates (Figure 1A), a proportion of which was associated with cell proteins (Figure 1B). Co-incubation of MRC-5 cells with ^3^H-BMAA along with the protein synthesis inhibitor CHX significantly reduced the amount of radiolabel in the protein fraction (Figure 2). CHX inhibited incorporation of the protein amino acid ^3^H-leucine into MRC-5 proteins to the same extent as ^3^H-BMAA (Figure 2) suggesting that ^3^H-BMAA was also incorporated into proteins by a protein synthesis dependent mechanism. ^3^H-BMAA was also found to be protein-associated after incubation with HUVEC and SH-SY5Y; this process was again found to be protein synthesis dependent and was inhibited by CHX (Figure 2).

**Figure 3:**
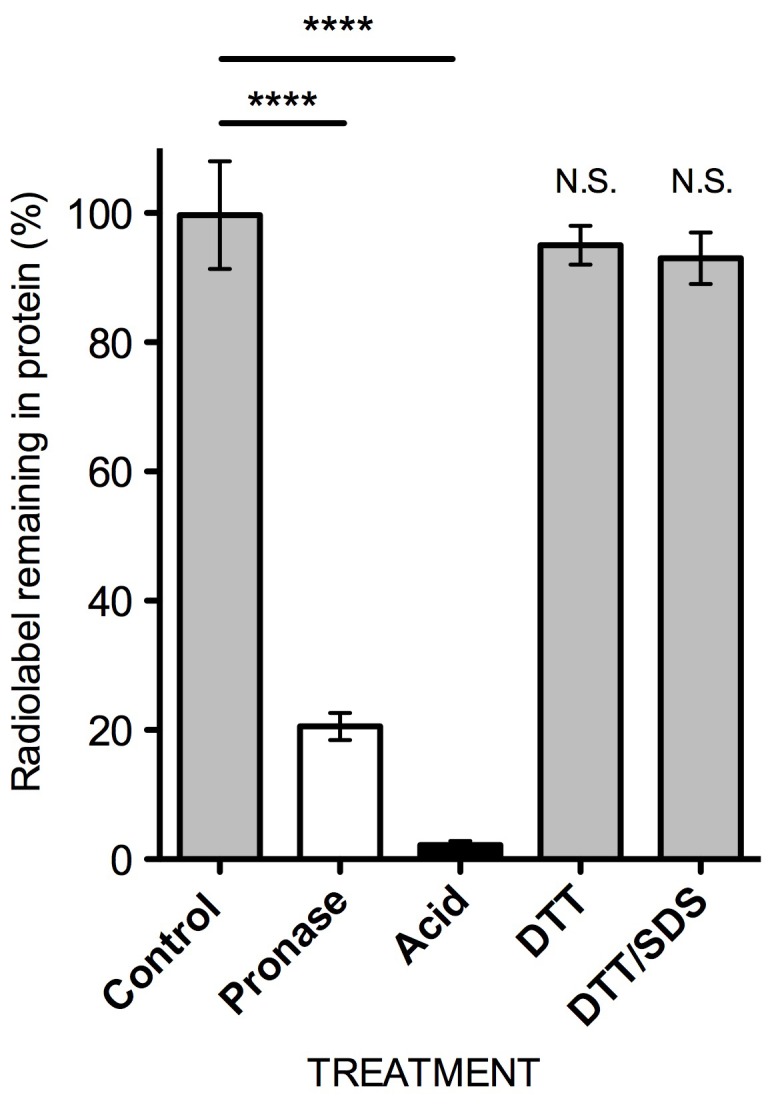
Recovery of radiolabel from cell proteins following incubation of cells with ^3^H-BMAA. SH-SY5Y cells were incubated with ^3^H-BMAA, cell proteins precipitated by TCA (10%) precipitation and the amount of radiolabel released from proteins (i.e. not TCA precipitable) after incubation with DTT or DTT and SDS quantified. Percentage of radiolabel remaining in proteins following treatment is expressed as a % of buffer alone. Cell proteins were also incubated with pronase or HCl and the release of radiolabel quantified relative to that of buffer alone (for Pronase) or water (for Acid). Values are mean +/− SD, *P*<0.0001 using Student’s two-tailed t-test, three independent tests (n = 3).

To further examine the association between BMAA and cell proteins, we examined the ability of a range of treatments to release radiolabel from cell proteins. Radiolabeled cell proteins were generated by incubating SH-SY5Y cells with ^3^H-BMAA for 24 hours. The radiolabel could not be removed from the isolated cell proteins by incubation with a 100 fold molar excess of the reducing agent DTT or by heating with the detergent SDS in the presence of DTT (Figure 3). Release of radiolabel required cleavage of peptide bonds by either acid hydrolysis or proteolytic cleavage of peptide bonds with pronase (Figure 3).

### BMAA Incorporation was Inhibited by l-serine

**Figure 4:**
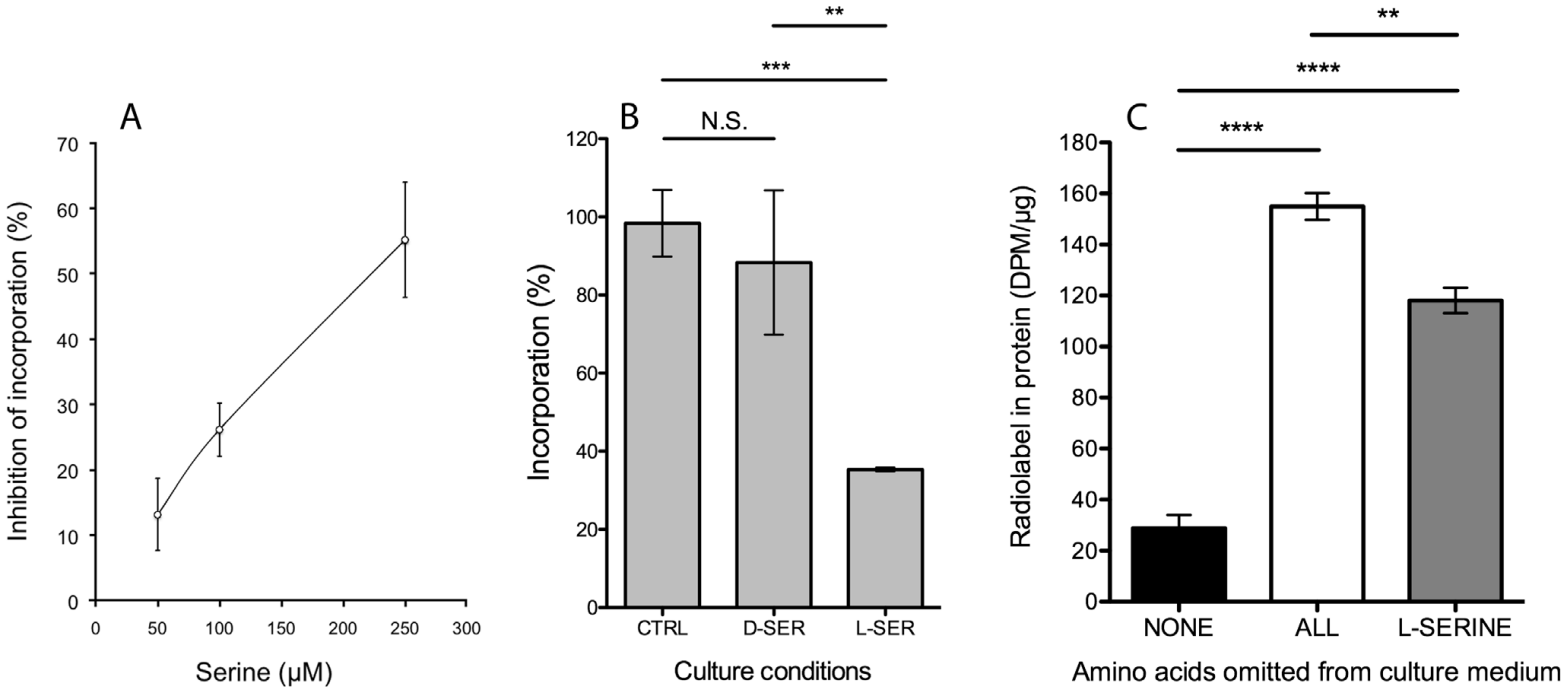
Inhibition of incorporation or radiolabel into cell proteins by l-serine. Panel A, Incorporation of radiolabeled BMAA was inhibited by l-serine in a concentration dependent manner. Panel B, d-serine (D-SER) had no significant impact on the incorporation of BMAA (NS, *P* = 0.4419). l-serine significantly inhibited the incorporation of BMAA compared to control cells (CTRL, ***P = 0.0002) and d-serine (D-SER, **P<0.01). Panel C, there was a significantly (****P<0.001) greater incorporation of BMAA when all protein amino acids were omitted from the culture medium (ALL) compared to when none were (NONE). When only l-serine was omitted (l-SERINE) incorporation was restored to approximately 80% (****P = 0.0009). Student’s two-tailed T-test, values are mean +/− SD for three independent experiments (n = 3).

To determine if a specific protein amino acid was being replaced by BMAA, we examined competition between all 20 protein amino acids and ^3^H-BMAA for incorporation into cell proteins. We found that incorporation of ^3^H-BMAA into cell proteins was inhibited in the presence of l-serine in a concentration-dependent manner (Figure 4A). d-serine, which cannot be used in protein synthesis by mammalian cells, did not prevent incorporation of ^3^H-BMAA into protein (Figure 4B). Incubation of cells with ^3^H-BMAA in culture medium which contained all 20 protein amino acids (“NONE”) at 400 µM each, greatly reduced the amount of radiolabel in the protein fraction relative to amino acid-depleted culture conditions “ALL” (Figure 4C). When l-serine alone was omitted from the amino acid mixture, “L-SERINE”, incorporation of BMAA into proteins significantly increased (Figure 4C).

### BMAA Released by Protein Hydrolysis was Positively Identified with LC/MS/MS

**Figure 5:**
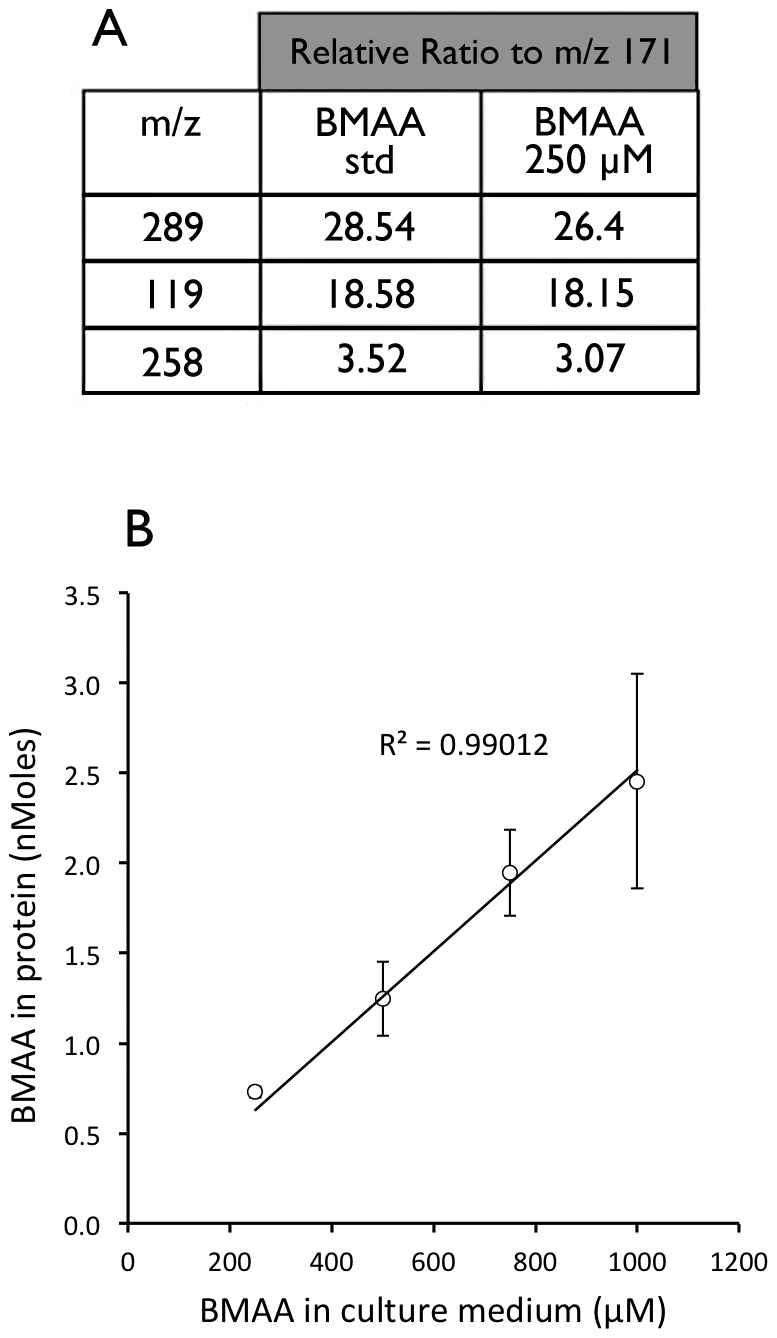
Identification of BMAA in cell proteins using tandem mass spectrometry. Panel A, a BMAA standard and a hydrolyzed protein sample (from MRC-5 cells treated with 250 µM BMAA for 24 hours) were run on triple quadrupole LC/MS/MS. Retention times, unique daughter ion (*m/z* 258), and ratios of *m/z* transitions during collision-induced dissociation of the *m/z* 459 parent ion matched those of an authenticated BMAA standard. Panel B, linear (r^2^ = 0.99012) recovery of BMAA from the hydrolyzed proteins as determined by triple quad LC/MS/MS. Values are mean +/− SD from three independent incubations (n = 3).

To further confirm that BMAA was present in cell proteins, we incubated MRC-5 cells with a range of concentrations of BMAA and analyzed the hydrolyzed cell proteins by tandem mass spectroscopy on a triple quadrupole LC/MS/MS. Retention times, unique daughter ion (*m/z 258*), and ratios of *m/z* transitions (*m/z* 459 to *m/z* 171 relative to *m/z* 459 to *m/z* 289 and *m/z* 459 to *m/z* 119 respectively) during collision-induced dissociation matched those of an authenticated synthetic BMAA standard (Figure 5A). Incubation of MRC-5 cells with increasing concentrations of BMAA, recovery via acid hydrolysis and triple quadrupole LC/MS/MS analysis produced a linear recovery of BMAA from the hydrolyzed proteins (r^2^ = 0.99, Figure 5B).

### BMAA Induced Autofluorescence was Abrogated when Co-incubated with l-serine

**Figure 6:**
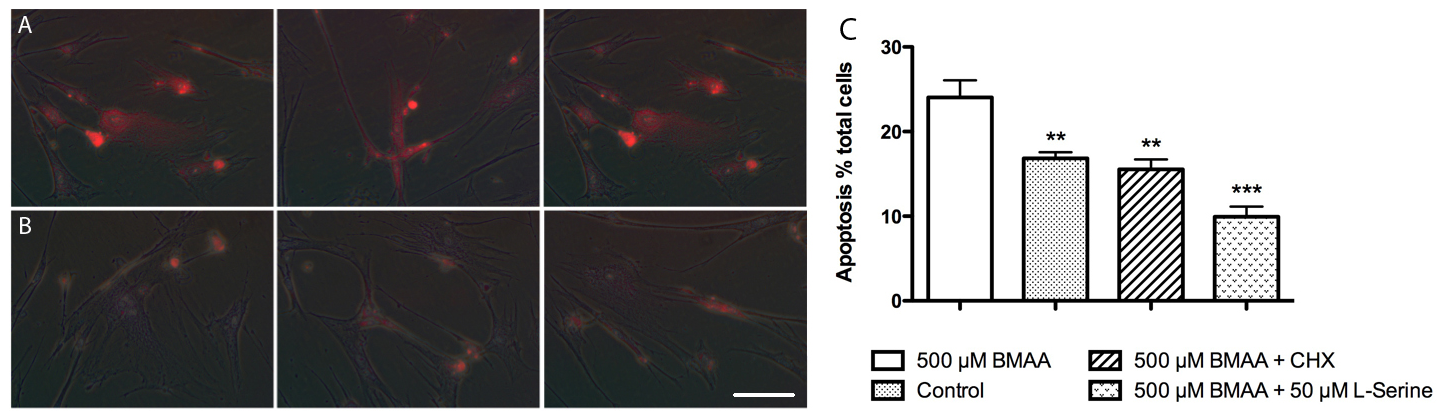
Incubation with BMAA results in the formation of autofluorescent bodies and apoptotic changes in cells. Panel A, Autofluorescence was observed in the cytosol and perinuclear regions of MRC-5 cells incubated with 300 µM BMAA. Panel B, autofluorescence was reduced when cells were co-incubated with 300 µM l-serine. Cells were incubated in 6 well plates and showed consistent perinuclear and cytosolic autofluorescence of varying degrees of intensity in BMAA treated cultures. The overall intensity of the signal was reduced when cells were co-incubated with l-serine. Images are representative of at least 12 fields of view from triplicate cultures. Scale bar is 100 um. Panel C, incubation with BMAA induced significant (**P = 0.0044) apoptosis in SH-SY5Y cells compared to control cells, which could be abrogated when co-incubated with l-serine (***P = 0.0005) or CHX (**P = 0.002). Student’s two-tailed T-test, values are mean + SD from three independent incubations (n = 3).

Autofluorescence, indicative of protein aggregation, developed in cells incubated with 300 µM BMAA for 96 hours (Figure 6A) and this could be abrogated by co-incubation with an equimolar concentration of l-serine (Figure 6B). Autofluorescence was localized to perinuclear and cytosolic regions of the cell but appeared consistently across each well (Figure 6A). The absence of changes in PI binding or in LDH release indicated that MRC-5 and SH-SY5Y cells were not undergoing necrotic cell death (data not shown). However, apoptosis was present as indicated by increased staining for Annexin V (Figure 6C). Apoptosis could be reduced by co-incubation with l-serine and also by CHX, which suggests that apoptosis was triggered by BMAA incorporation into proteins (Figure 6C).

Taken together these four different lines of evidence suggest that incorporation of BMAA into cell proteins is a protein synthesis-dependent process, which can be inhibited by l-serine.

## Discussion

Protein misfolding, evidenced by the presence of aggregated and tangled proteins manifested by neuroanatomical abnormalities such as Bunina bodies, Lewy body inclusions, tauopathies, senile plaques, and neurofibrillary tangles, is increasingly viewed as an engine for disparate progressive neurodegenerative illnesses. Few environmental agents have been identified that can induce protein misfolding, subsequent protein aggregation, and apoptosis with the notable exception of prions which are believed to be responsible for Kuru, Creutzfeldt-Jakob Disease, or Bovine Spongiform Encephalopathy. We here report that unusual amino acids can, in a similar manner, induce protein misfolding through misincorporation for one of the regular 20 canonical amino acids.

Motor neurons, as a special class of post-mitotic cells, are particularly vulnerable to terminally aggregated proteins since they are unable to reduce the burden of protein aggregates by distributing them amongst daughter cells. This was evident in a study in which neurodegeneration was the primary pathology in a mouse with a minor translational proofreading defect [2]. Vulnerability may also be enhanced in motor neurons of significant length where axonal transport may be compromised by protein aggregates. In addition, adjacent cells may be impacted. Luk et al. [24] found that injection of synthetic misfolded α-synuclein into mouse brains cells results in a neurodegenerative cascade of misfolded proteins in adjacent cells. If misfolded proteins resulting from misincorporation of non-protein amino acids can be transmissible within the brain through a prion-type mechanism [25], it is possible that a neurodegenerative cascade could be triggered.

Our finding that BMAA can be misincorporated for serine in human proteins raises the possibility that such misincorporation results in neurodegenerative illness. Phosphorylation of proteins typically occurs on serine, threonine, tyrosine, and histidine residues. Some serine sites such as tau serine residue 422 or TAR DNA-binding protein 43 (TDP-43) serine residues 409/410 have been identified as being key to neuropathologies [3], [26]. BMAA misincorporation at such sites may be particularly detrimental to proper protein functioning. *In vivo* confirmation of BMAA-induced protein misfolding and aggregation could be obtained if incipient tauopathies, Bunina bodies, or other protein inclusions were to be detected in non-human primates fed BMAA.

Exposure of human beings to BMAA has been documented on Guam where the presence of protein-associated BMAA in cycad flour was first observed by Polsky [27]. Protein-bound BMAA was subsequently shown to be 50–100 times more abundant in flour than the free form of BMAA [13], [28]. Protein-associated BMAA has since been found in cyanobacteria that are endosymbionts in the coralloid roots of cycads, animals that forage on cycad seeds, and post-mortem brain tissues of Chamorro villagers who died of amyotrophic lateral sclerosis/Parkinsonism dementia complex (ALS/PDC) as well as North American patients who died of ALS and Alzheimer’s disease [13], [29], [30].

We found the misincorporation of BMAA into human proteins to be a protein-synthesis dependent process in primary human endothelial cells, and in human fibroblast and neuroblastoma cell lines since it was significantly reduced when protein synthesis was partially inhibited by the addition of CHX (Figure 2) or when the concentration of the protein amino acid l-serine in the culture medium was increased (Figure 4A). Further, d-serine, which cannot be used in protein synthesis by mammalian cells, had no effect on the incorporation of ^3^H-BMAA (Figure 4B). Previous studies have demonstrated that acid hydrolysis was required to disassociate BMAA from proteins [13] and we here report that BMAA can also be released from proteins by proteolytic cleavage (Figure 3), providing further evidence that BMAA is peptide bonded into the amino acid chain of proteins and peptides.

BMAA and other non-protein amino acids can mimic one of the 20 canonical amino acids and become mistakenly incorporated into proteins [6]–[8], [19], [21], [31]. A high level of fidelity in protein synthesis is maintained because of the very low error rate on the part of tRNA synthetases when selecting the correct protein amino acid for esterification to the cognate tRNA [1]. tRNA synthetases however are less efficient at discriminating against certain non-protein amino acids of a similar, size, shape and charge to a protein amino acid [31].

Aggregated proteins are characteristic of neurodegenerative disease, hence they are sometimes known as “proteinopathies” owing to the deposition of aggregates of tau, α-synuclein, huntingtin, superoxide dismutase-1, or TDP-43, which characterize such human neurodegenerative disorders as frontotemporal degeneration, Parkinson’s disease, Lewy body disease, Huntington’s disease, and ALS. Our treatment of fibroblast cells with BMAA resulted in the development of cellular autofluorescence characteristic of protein aggregation (Figure 6A). We have previously demonstrated that insertion of a non-protein amino acid into a protein can result in protein misfolding and loss of aqueous solubility [32] as well as the generation of *in situ* autofluorescent material [21]. Incorporation of BMAA into proteins *in vivo* might therefore be capable of generating misfolded proteins that can ‘seed’ further protein aggregation in cells [33].

BMAA incorporation into proteins was found to induce apoptosis in neuroblastoma cells *in vitro*, an effect previously reported with other non-protein amino acids [23]. Incorporation of BMAA into protein is also consistent with the dysregulation of cellular protein homeostasis and ER stress reported by Okle [34]. Apoptosis and ER stress are inextricably linked to neurodegeneration and the gradual accumulation of BMAA-containing protein aggregates in motor neurons could account for the chronic toxicity reported following BMAA exposure which is preceded by a long latency period [35].

Following a single i.v. bolus injection of ^14^C-labelled BMAA into the femoral vein of rats, Xie [36] found that BMAA persisted in the brain with a time course consistent with ‘trapping’ by cerebral proteins. 1.5 hours after injection, free BMAA accounted for more than 95% of BMAA in brain tissue but declined steadily over the next 7 days while levels of protein-associated BMAA remained relatively stable and accounted for around 80% of the BMAA in the brain after 7 days. These studies are consistent with BMAA being incorporated into proteins by protein synthesis and an inability by neuronal cells to degrade these proteins. It is feasible that protein-incorporated BMAA could accumulate in specific regions of the brain as was evident in the study of Karlsson [14] and this could relate to differences in the rate of uptake of free BMAA, the rate of protein turnover and the availability of l-serine in different brain regions.

BMAA induces peptide changes in neonatal mice, with demonstrable cognitive deficits in the adult mice [37] and this is similar to deficits reported in honeybees [38]. Rats exposed to BMAA produce neuropathologies similar to ALS [39]. Electron microscopy of the spinal cord of BMAA-treated rats revealed a number of changes that are consistent with incorporation of a non-protein amino acid into proteins including elevated caspase 3 [23], apoptotic changes [23], swollen cristae and general disorganisation of mitochondria [40].

Whilst the acute effects of BMAA on neuronal cells [41], [42] and glial cells [43] have been clearly elucidated, epidemiological studies of human health impacts of chronic BMAA exposure are nascent. Our findings that BMAA can be misincorporated into human proteins *in vitro*, with subsequent protein misfolding may shed new light on reports of putative associations between human exposure to cyanobacteria which produce BMAA and higher than expected incidence rates of ALS. These associations have been reported in residents of New England who lived near lakes subject to frequent cyanobacterial blooms [44], among consumers of shellfish in Chesapeake Bay [45], soldiers deployed to the Gulf War between 1990–1991 [35], and in the Western Pacific ALS foci including the indigenous people of Guam [13], [29]. Proposed longitudinal surveys [46] may provide a rigorous test of such putative spatial associations.

The finding that l-serine can block the misincorporation of BMAA into human proteins may prove useful in designing novel therapies for ALS.
